# Collectanea

**Published:** 1846-06

**Authors:** 


### Collectanea.

*Hemorrhagic Diathesis—Ten Days Hemorrhage after the Extraction of a Molar Tooth.*—Dr. Clay of Manchester reports, in the *Medical Times,* the case of a boy, aged fourteen, who applied to a druggist to have a tooth extracted on the 17th of Nov. last. Considerable hemorrhage occurred immediately after the operation. On the following day, (Nov. 18th,) the hemorrhage continued in increased quantity. The family became alarmed, and the druggist having failed to stop the flow of blood by plugging the parts with lint moistened with the tinct. myrrh, and other styptics, I was sent for. I found the boy bleeding freely, with the whole interior of his mouth cauterised by the nitrate of silver, which had been previously applied to such an extent that much unnecessary pain and inconvenience were caused in the after-treatment. The plugs applied at the time of my arrival not checking the flow of blood, I removed the whole, and had the mouth well washed out, and, with a strong light, ascertained, as well as I could, the injury the parts had sustained. I found that the third molar tooth of the upper jaw, on the right side, had been extracted, and the soft parts extensively lacerated, not only forwards but backwards, almost to the fauces, and also a deep laceration in the cheek; in addition to this extensively lacerated surface, the alveolar processes had been broken down by the violence used. As might be expected, blood poured out from different parts of the injured surface, of a decidedly arterial character.

Before detailing the treatment of this case, I will give some particulars of the family to which the boy belongs, in many of the members of which the hemorrhagic tendency is much developed; this is entirely confined, however, to the maternal side, and the facts were only recollected casually during the treatment.

The boy's uncle (William) had a tooth drawn, which bled freely for some days. A second uncle (Josh.) had an injury of the head, which bled for some days, and only yielded to very energetic treatment. A third uncle, when about the same age as the present patient, had a tooth extracted, which bled profusely for eleven days. An elder brother had a tooth extracted, which bled seven or eight days. Another brother had a cut finger, which bled for two or three days, and a tooth extracted, which bled three or four days. Lastly, the subject of the present case, when about four years old, had a tooth extracted, which bled four days; when eleven years old, he cut his thumb, which bled five or six days; when about twelve years old, another tooth was extracted, which bled two or three days; thus, previous to the present accident, there had been no less than nine cases of prolonged hemorrhage from injury occurring to members of this family. No attention, however, seems to have been paid to these facts, which had been so far forgotten, that no care was used to select a competent person to perform the present tooth extraction.

After having ascertained the extent of injury, as before stated, I replaced the plugs, saturated with the tinct. fer. mur., adapting them with great care to the injured parts. A well fitted piece of cork was then inserted in order to increase the pressure, and the mouth was closed, as the hemorrhage appeared to be checked. I left the patient, but was sent for again in three or four hours, and found him bleeding quite as freely as at first. Considering that it arose from deficiency of pressure in some part of the plugs, I determined on removing them. I used the tinct. fer. mur. with a few drops of muriatic acid for the second dressing, and replaced the plugs and subsequent pressure with every care, but the extent of laceration was such, that it was impossible to produce pressure on all the partsat once. A second time all appeared quiet, but in a few hours the bleeding was as active as ever. Some particulars of the family tendency were now told me, and I determined on using the matico; first, by covering the parts with the powder, supported by pads of dry lint, adding a cork pad, and fastening the jaws close together, and, after a trial of some hours, without benefit, by using the moistened leaf of the plant with no better effect. The powdered secale cornutum was next tried, still the wound bled as freely as before. It now became manifest that the constitution was at fault, in addition to the great injury by laceration, and it was also equally evident that unless some very determined course was taken, the boy must sink. I was averse to the use of the actual cautery, and equally so to tying the external or common carotid, but these means, with an attempt at constitutional treatment, were the only ones left. I determined on taking another opinion before I proceeded any further. Shortly after Mr. Wilson met me in consultation, and it was agreed to give the matico another trial, but with no greater success than on the former occasion. Active purgatives were given, and, at a second meeting, it was determined to try the old fashioned remedy "*Ruspini's styptic,*" not only externally, but in two tea-spoonsful doses every three or four hours; no good, however, appeared; the matico was then placed on pads, moistened with Ruspini's styptic, but still the bleeding continued. At every opportunity, when new dressings were applied, as much nourishment as possible was poured into the system, consisting of milk, eggs, strong broths, jellies, &c. Ice was applied about the neck and throat, and whenever an opportunity offered, the mouth was washed out with iced water. Things proceeded thus to the end of the sixth day, with little or no abatement of the hemorrhage, the boy's countenance became blanched, with a wandering and anxious expression. It was now determined to use the actual cautery; this was freely and frequently applied, but not the slightest good resulted from it; it was no sooner burned in one place where the bleeding seemed most frequent than it burst out at another, and when that again was seared, the former eschar (if it could be called one) gave way and bled as freely as before. This cruel mode, which the boy bore with the most heroic fortitude, was finally abandoned as useless, if not productive of mischief by enlarging the abraded surface. It was equally evident that any operation of tying the artery would only be adding a fresh wound to contend with. Constitutional treatment alone remained. A consultation was held by myself, Mr. Ainsworth, Mr. Thorpe and Mr. Wilson, when it was determined to give internally the following mixture:—

℞ Plumbi. super. acet. ʒ ss; Acid. acet. dil. ℥ ss; Syr. Rhead. ℥ ss; Mist. camph. ℥ v. M. ft. mist. ℥ vi. Sumat æger coch. magn. duo omni tertia hora.

Thus, five grains of the acetate of lead were given every three hours, or nearly so, in addition to which, pads saturated with the liq. plumbi. diacet. were applied to the bleeding surfaces, with as correct a pressure as possible. Twenty-four hours passed under this treatment without improvement. At a second consultation with the gentlemen above named, in addition to whom Mr. Bamber, a retired practitioner, and formerly the medical attendant of the family was present, it was agreed to persevere with the acetate of lead mixture until the day following, continuing also the pads of lint saturated with the liq. plumbi. diacet., covering the pads also with finely pulverised matico. On the following morning, much to the satisfaction of all parties, the bleeding was checked; this was the tenth day, and up to this time he had taken nearly a drachm of the acetate of lead. The boy began to complain of pain in the stomach and head; it was therefore evident we could not proceed further with the acetate; and as the bowels had not been moved since its exhibition, it was determined to substitute the sulphate of soda, which the American physicians speak so highly of. A saturated solution was ordered to the extent of a wine-glassful, every four hours, and in a very short time the bowels were acted upon. The bleeding was perfectly arrested from the tenth day, great care being taken for some time in removing the pads, and which were for several days moistened with the liq. plumbi. diacet. I have omitted to mention that the head was shaved when the constitutional treatment commenced, and that evaporating lotions were kept constantly upon it. From the tenth day, the boy very gradually recovered, though extremely reduced. He is now (December 29th) comparatively well, and all the soft parts of the mouth are perfectly healed.

This case is an instructive one, as it serves to show how very little dependance can be placed on styptics, or, indeed, on any local application; in cases of hemorrhagic diathesis, constitutional treatment alone is the best, indeed the only mode by which we can hope for permanent advantage. I have seen cases reported, where abstraction of blood has been practised, but for what end I am utterly at a loss to understand, except that of ascertaining (by analysis) if the blood be deficient in fibrine; but surely it is unnecessary to do this, as it does nothing towards the cure, whereas the abstraction of blood adds to the difficulties of the case. If such cases were difficult of diagnosis, the test might be barely justifiable, but they are not; therefore, to take from the mass of an already too impoverished system, cannot, in my opinion, be defended on any grounds. The actual cautery, I think, is equally to be condemned, fresh wounds should never be made, and this applies also to the tying of any artery, the common carotid being the only one likely to effect a check in hemorrhage from the gums; yet, as that has been done by the first English surgeons, and has failed, it ought never to be again resorted to. It will, perhaps, be unnecessary to inquire how the acetate of lead or the sulphate of soda effected the object sought in this case, *that is,* to restore the principle of coagulation to the blood. That the blood is deficient in fibrine will, I think, be generally admitted, but there is nothing in half a drachm of acetate of lead, nor even in an ounce or two of sulphate of soda, to which can be attributed the formation of a new supply of fibrine in a few hours. These means must act on some other principle, perhaps by abstracting a considerable portion of the serous part of the blood, the lessened mass which remains, being more in proportion for the object of coagulation than heretofore, accomplishes the wished-for result. At any rate, it is fortunate to have two such remedies as the plumbi. sup. acet. and the sulphate of soda, inasmuch as the first cannot be used long without producing very distressing symptoms, pains in the head and stomach, and obstinate constipation. But by pushing it rapidly in large doses until these symptoms come on, and then substituting a strong solution of the sulphate of soda, which, while it opens the bowels, and relieves the pains produced by the lead, has an equally specific power in improving the tendency to a healthy coagulation, we possess a good means of treating cases of this nature.

*Necrosis of the Lower Jaw.*—At a recent meeting of the *Medical Society of Emulation*, in Paris, M. Depaul related the case of a girl aged eight years, who had, previously to her applying to him, enjoyed good health, became affected, in March, 1845, with a severe pain in the left side of the lower jaw, the pain being apparently due to decay of the two first temporary molar teeth. The pain shortly afterwards subsided, but was reproduced soon after the cessation of a slight eruption of scarlatina. A swelling then showed itself in the lower jaw, and was followed by an abscess which opened in the mouth at first, and created, a little later, ulceration beneath the angle of the maxilla. The abundance of suppuration weakened the patient considerably, and hectic fever and nocturnal perspirations also contributed to the impoverishment of her constitution. Under those circumstances, M. Depaul's attendance was requested, and he found, on examination, (July 29,) that a loose fragment of denuded bone could be distinctly felt with a probe through the cutaneous ulcer, and with the extremity of the little finger introduced into the orifice situated within the mouth. Having slightly enlarged the inner opening of the abscess, M. Depaul removed a dead fragment which had formed the angle of the jaw, the greater part of its ramus, and one-third at least of its body. The necrosed structure contained a tooth belonging to second dentition. Since the operation the condition of the patient is very much improved, the febrile symptoms have disappeared, and the orifice of the abscess secretes no more pus.

*A Case of Tetanus Successfully Treated.*—Mr. Pushaw states in the Lancet, that he was consulted by a patient who was laboring under a slight stiffness of the neck; had pain and difficulty on moving his head, legs, &c. I inquired if he had sustained any injury. He said he had not. Ordered a smart purgative of calomel and jalap, a warm bath for twenty minutes, and an antispasmodic mixture of sulphuric ether and tincture of opium. Towards evening the rigidity of the muscles of the head and neck increased, attended with difficulty in swallowing, tightness about the chest, and inability to move the jaw; the teeth were firmly set, almost every muscle quite rigid, and the spine bent into an arch, leaving no doubt as to the nature of the case. The boy still denied having received any injury. Ordered the spine to be well rubbed with a strong liniment, and the warm bath to be repeated. Next day the boy told me that, on the previous Saturday, (18th Oct.) while stooping at play, a person struck him with a stick over the loins. He did not seem to have felt it much at the first, but the following day said that he felt as if unable to move his legs. Examined the spine, no mark of any injury, but he complained of a slight tenderness over the lumbar region of the spine. Ordered nine ounces of blood to be removed by the cupping-glass from the spot where he seemed to feel most pain. No relief. Next day ordered a dozen leeches to the same place, and a large blister along the spine. The symptoms not relieved. The warm bath as before. Unloaded the lower bowels by a saline enema, and gave an opiate. Next day to administer two grains of calomel every three hours, with a view to affect the mouth; continued for forty-eight hours, and, although aided by the copious use of strong mercurial ointment rubbed in inside the thighs, failed in producing the desired effect. Leeches again to the spine. Acted on the bowels by a dose of croton oil, and then administered the oil of turpentine by the mouth, and as an enema. After a due trial this failed. Ordered a blister to the lumbar region of the spine, and two grains of the muriate of morphia to be sprinkled over the blistered surface, and gave the solution in large doses, and at short intervals, but without the least benefit, Several medical friends attended with me, and the above means were persevered in for three weeks. The patient was rapidly sinking from want of nourishment, &c. A physician, who had seen the case, kindly sent me The Lancet, of 22d March, 1845, in which there is a case recorded by J. W. Stapleton, Esq., "On the Administration of Intoxicating doses of Alcohol in Tetanus;" but the result not being favorable, I declined this mode of treatment. I had thought of the Indian hemp, but the conclusions of Dr. Lawrie, of Glasgow, after having tried it in twenty-six cases, gave me little confidence in it.

I was, however, inclined to try belladonna, or arnica, when the friends desired a consultation with a French physician. He advised a trial of the hydropathic treatment. Many difficulties presented. The patient was at a distance from my house; I could not receive him there, nor had I liberty to treat him at the public hospital, and the system, to be fairly tried, required more favorable circumstances than those in which the boy was placed. However, I at once enveloped him in a linen sheet, well wrung out of cold water. Over this I placed three or four good blankets, &c., so as to exclude the air, and prevent evaporation. Kept the patient in this condition for an hour, by which time the temperature of the sheet was 100° Fahr. The coverings were removed, and the patient plunged into a cold bath, rubbed quite dry, and enveloped in dry blankets for six hours, during which time he perspired very freely, and slept soundly, and said he "felt quite *slack*." Repeated the cold bath while in a state of profuse perspiration, and, after an interval of an hour, the wet sheet and subsequent cold bath. This was repeated every six hours, and after twenty-four hours, the jaw relaxed a little, and the spine became less bent. I now placed him under the douche for three minutes, the water falling from a height of twenty feet, and in a stream of one inch and a half diameter. Dried and enveloped in the blankets as before. Able to open the mouth a little. Ordered the sheet and cold bath as before, and ten days from the commencement of the treatment, every symptom yielded, and the boy is now quite well. It cannot be said that "cold water" is a new remedy in such cases, for Dr. Cullen, in his "First Lines of the Practice of Physic," published in 1796, p. 341, observes, "The administration of it is sometimes by bathing the person in the sea, or, more frequently, by throwing cold water from a bason or bucket upon the patient's body, over the whole of it. When this is done, the body is carefully wiped dry, wrapped in blankets, and laid in bed, and, at the same time, an opiate is given, and, by repeating this, the patient is often quickly cured." Believing the douche to be a decided improvement on the bucket of water, and the wet sheet on the opiate, (for its effects are most soothing,) I would crave for this case a corner in your valuable journal. My medical friend, who sent me The Lancet containing Mr. Stapleton's case, observed, that in such diseases, every contribution is valuable. With this thought, I enclose it to you.

---

*Necrosis, &c., from the Pressure of the Dens Sapientiæ.*—M. Monod, at a meeting of the Academy of Sciences, related some curious cases of this disease, which had come under his care, one of which arose from the pressure of the wisdom tooth on the root of the second molar, occasioned much inconvenience and suffering. A gentleman became deaf in both ears without any evident cause; at the same time he complained of severe rheumatic pains in both rami of the maxilla. No tooth was decayed, but the localisation of the pain in the vicinity of the second molar induced M. Monod to remove it. After the operation, the symptoms immediately disappeared. On examination of the fangs of the extracted tooth, they were found to be worn and dovetailed by the pressure of the wisdom tooth.

A man, aged 80, whose teeth had all fallen away, presented, near the angle of the maxilla, a fistulous opening, kept open by the presence of necrosis. On examination of the mouth, a tumefaction was noticed in an alveola: the part was carefully probed, a tooth was discovered and extracted, and the fistula closed; it was the wisdom tooth.—M. Guersant: A girl, ætat. 16, was brought to M. Guersant, with a fistulous opening in the anterior region of the neck, the consequence of an abscess. On examining the teeth, he found that one of the inferior canine teeth was discolored, and that he could introduce a probe between the tooth and the alveola: the tooth was removed and the fistula cured without delay. M. Chassaignac remarked that in preparing the lymphatics of the head and neck in young subjects, he had often met with an anatomical change of structure, of some interest to the present debate. The tooth examined in the alveola presents no alteration; but the maxilla being completely denuded, is found perforated with several small holes in the neighborhood of the root of the tooth. If this diseased lamina of bone be removed, the fang of the tooth is generally found diseased and surrounded with pus. This intra-alveolar caries must often escape observation during life, and is, doubtless, in many instances, the cause of the enlargement of the maxillary glands, and of certain purulent collections of the same region.

---

*On Necrosis of the Jaw Bones.*—At a recent meeting of the Medical Society of Nuremberg, Dr. Heifelder spoke on necrosis of the jaw, first mentioned by Dr. Lorinser, of Vienna, and lately occurring in Vienna and Nuremberg, among individuals employed in the manufacture of matches, and attributable to the inhalations of phosphoric vapors. Drs. Sicherer and Blumhardt then stated that similar cases had fallen under their notice in Ludwigsburg and Stuttgart, especially in the latter place, in children. Professor Dietz remarked, that up to this time the disease had been found to affect only the female workers of one and the same manufactory. Much had been ascribed to the influence of the workshop, through which an excessive draft of air passed, and the laborers, on account of the high temperature maintained, wore very light clothing. Similar cases, however, have since been noticed in other places, and it appears probable that the disease arose from the phosphorous vapors with which these localities are impregnated. This opinion seems the more likely, as many patients had not been exposed to the influence of the chloride of potassium, (chlorate of potassa.) It cannot be overlooked, however, that during the time the manufactory of Nuremberg has employed phosphorus free from arsenic, the number of patients has become less. Professor Fuchs remarked, that arsenical vapors are by far less injurious to health than has been hitherto supposed. This is proved in the case of the mines in the Hartz mountains. Professor Scherer objected to allot any share in the production of the above disease to arsenic, as it is not so volatile as phosphorus. The president said that the effect of arsenic is frequently seen gradually to disappear. Dr. Roser had observed a similar evil affecting male individuals. This discussion having been brought to a close, Professor Dietz and Dr. Geist introduced three patients who had been under their treatment. These cases were the more interesting as, in two of them, the disease was in its first stage; and several specimens of the lower jawbone, partly extracted by an operation, and partly removed after death, were well adapted, by their pathological changes, to show the termination of the disease.

*Diseases of the Tongue.*—Mr. Lawrence observes that the most frequent forms of disease in the tongue are ulceration, generally superficial, sometimes more deeply seated; swelling and thickening of the mucous membrane; swelling and induration of the substance of the organ. Ulceration often exists in conjunction with swelling of the mucous membrane, and with induration of the lingual substance. The more formidable diseases of the tongue are syphilitic or cancerous; the former being by far the most numerous. Disorder of the digestive organs is sometimes the source of the mischief, giving rise to affections, which are usually superficial, but sometimes of more serious character.

Several cases of these diseases, are related in the course of his lectures. The first is one of circumscribed swelling and induration, with deep ulcerated fissure. The patient was a married woman, twenty-six years of age, and her previous history, although she denied having had syphilis, yet presents several suspicious circumstances, such as an eruption over the body, simultaneously with ulcerated sore-throat, and repeated relapses of the lingual disease. When admitted into the hospital there was a circumscribed swelling of oval shape, the size of a large filbert, on the right side of the tongue: it was of pink color, firm, but less hard than scirrhus. In its long axis and whole length, there was a deep ulcerated fissure, the edges of which were irregular but not everted, and the surface was not foul. It did not cause much pain; and there was no affection of the lymphatic glands. The treatment consisted of the exhibition of mild mercurials internally, and the use of an alum wash to the mouth. The patient was discharged four weeks after her admission, the swelling and induration having nearly disappeared, and the ulceration being cicatrised. In the next case, that of an unhealthy looking girl, there was a circular excavated ulcer, the size of a shilling, on the dorsum of the tongue. It had a greyish surface, with raised edges, and considerably indurated basis. The disease commenced with a hard patch on the tongue, in the situation of the ulcer. The girl strongly denied any possibility of syphilitic taint, and did not present any symptoms of that disease, notwithstanding which, the disease is classed among the syphilitic affections. She was discharged cured in about a month. The treatment consisted of the internal exhibition of hydrargyrum cum creta, with sarsaparilla.

The third case was clearly shown by the history, symptoms, and treatment, to be syphilitic. The man had on the right side of the tongue, a rough ulcerated surface with everted edge, the size of a shilling, and at the posterior part of the tongue, on the same side, the mucous membrane, over a space the size of a shilling, presented a smooth red surface, free from papillæ, in the centre of which there was a deep foul ulcer, with yellow surface, and hard circumference. The internal use of the hydrargyrum cum creta, in small doses, three time a-day, removed the hardness in a few days, and at the next report, twelve days afterwards, the ulcerations were nearly cicatrised. The patient then left the hospital.

The fourth case is one of phagedenic ulceration of the tongue and gums, and also of the trunk and legs, the result of the absorption of the venereal virus, eight years previously. The general health was much reduced. The hydriodate of potash, with sarsaparilla, was used for the first fortnight, when quinine was substituted for it. The local applications were the yellow lotion and poultices, to the sores, and the linimentum æruginis to the tongue and gums. The man had also meat diet and porter; at the end of six weeks, the ulcers on the body were healed, and the tongue and gums were quite well. He was then made an out-patient.

In the fifth case there was a phagedemic ulcer of the right eye-brow, with a large ulcer on the tongue, and enlargement and induration of both testes, with effusion of fluid into the left tunica vaginalis. The patient was treated by iodide of potassium in decoction of sarsaparilla, and afterwards with mild mercurial preparations. At the end of nine weeks, he was discharged, the ulcers being soundly cicatrised, the tongue free from pain and moveable, and the testes their natural size and much softer.

The sixth and seventh cases, instances of syphilitic ulcerated fissure, clearly indicate the advantage of a mercurial treatment. The eighth case as clearly demonstrates the utility of a mercurial course, in enlargement and induration of the tongue. The case was one of constitutional syphilis. Mr. Lawrence remarks that these cases do not exemplify the most common form of the disease, when of syphilitic origin. We see more frequently ulcerations, generally of small size, along the edges of the tongue, sometimes with greyish surface, superficial or a little excavated, sometimes with slightly raised edge. Not uncommonly, there is a thickened, raised and fissured state of the mucous membrane, sometimes combined with superficial or deeper ulceration. This thickened mucous membrane, may be either redder than natural, or paler; in the latter case, the epithelium is thickened and opaque. The dorsum of the tongue sometimes presents red patches, in which the mucous membrane is denuded of its epithelium, but is perfectly smooth, and not ulcerated. These may continue for a long while, getting better and worse, particularly if neglected. These diseased appearances are occasionally combined in the same case. In one instance in which Mr. Lawrence was consulted, and where there was decided evidence of a syphilitic cause, the surface of the organ, in its middle portion, and over more than half its extent, the dorsum and edges being included, was smooth, the epithelium being white and opaque, as if this part had been ulcerated, and partly having a raw appearance. There were three or four superficial ulcers, with grey surface, at the edges and tip, and small superficial ulcerations of the lips, with chaps at the angles of the mouth. In this case, the iodide of potassium had always done good, but whenever it was left off, the disease relapsed. Small doses of the hydrargyrum cum creta, exhibited until severe ptyalism had been produced, effected a cure. The beneficial influence of mercury on ulcers of the tongue, mouth, and throat, has always appeared to Mr. Lawrence, to afford the most unequivocal evidence of its peculiar antisyphilitic virtues; for while the sound mucous membrane is inflamed and ulcerated by the action of the remedy, the contiguous venereal ulceration, is seen to be altered in character, and healing rapidly. The enlargement and induration of the tongue, is a much less common form of the disease. Mr. Lawrence has always found it give way satisfactorily, to the use of mercury, and he gives several cases, additional to the eighth, already mentioned, in illustration. In the most severe of these he can only report a decided improvement in the state of the tongue, as he is unacquainted with its subsequent progress.

The patient was twenty-five years of age. The right half of the tongue was enlarged so considerably, as to impede articulation, the swelling beginning at the back, and extending nearly to the apex; it was also indurated, but less so than in scirrhus. The morbid change embraced both the mucous membrane, and the muscular substance, extending nearly throughout the entire thickness of the organ. The surface was red and slightly tuberculated, presenting three or four superficial ulcerations on the more prominent points. In front of the indurated mass, and near to the apex of the organ, there was a separate lump, the size of a horsebean, with a small ulcer on the surface. The disease extended to the middle line of the tongue; the very margin of the organ, constituting the outward boundary of the enlargement, was healthy. There was no other affection of the mouth or throat. The history of the case showed the disease to be syphilitic, and the treatment was managed accordingly. When the mouth was much affected, the state of the tongue, as already stated, had decidedly improved.—*Abridged from the Medical Gazette.*

---

*Fungoid Tumor of the Lower Jaw.*—A case of this disease, is reported in the *Provincial Medical and Surgical Journal,* from the practice of Mr. Greenhow, at the Newcastle Infirmary. The patient was a farmer, sixty-two years of age, who was admitted with a large fungoid tumor on the left side of the face and neck, extending from the chin in front, to the angle of the jaw behind, and above from near the zygoma down to the thyroid cartilage, projecting very high externally, and being found with a lobulated surface in the mouth; it had an unhealthy livid appearance, especially at its centre, where there was an opening made by the puncture of a lancet two months before, out of which issued an abundant discharge of bloody sanies; the skin was adherent to the tumor for a considerable distance around the ulcer, but at other parts was free and healthy; the tumor had an irregularly soft elastic feel, was moveable at that part which was situated above the jaw, but the other portion was adherent to the lower part of the jaw, and to the structures below it, by firm and immoveable bands. The disease was apparently produced by a blow received on the part five months previously. The man was very much out of health, and the disease rapidly making progress. The operation of extirpation of the tumor was attempted, and partially practised; but owing to its great extent, and its connexion with the important parts in the neck, it was not completely removed. The man, however, did well after the operation, and left the infirmary much improved in health. There is, however, every reason to fear that a relapse will occur, and terminate fatally.. The heterologous production consisted of vascular cerebriform matter.

*Aneurism of the Coronary Artery, of the Lower Lip.*—A pregnant woman, aged twenty-three, presented a tumor of the size of a hazel-nut at the right side of the lower lip. The disease had lasted for some time, when it ulcerated by the pressure of the teeth; hemorrhage was the immediate consequence, and was suppressed by compression of the coronary artery. The tumor which had, up to the period of ulceration been the seat of pulsations isochronous with the pulse, ceased to beat; and its further progress was completely arrested, spontaneous cicatrisation having taking place. The reporter observed that this case, as well as the two others, proved a fact which had already, from experience, received abundant demonstration, viz. that aneurism is occasionally susceptible of spontaneous cure, but could not be considered as an argument against the prudent practice of tying the diseased vessel.—*Abridged from the Gazette Medicale.*

